# Rapid Implementation of Virtual Health in a Pediatric Neurology Practice During COVID-19

**DOI:** 10.1017/cjn.2020.241

**Published:** 2020-11-05

**Authors:** James Lee, Dewi Schrader, Cyrus Boelman, Linda Huh, Mary B. Connolly

**Affiliations:** Department of Pediatrics, Division of Neurology, The University of British Columbia, Vancouver, British Columbia, Canada; BC Children’s Hospital, Vancouver, British Columbia, Canada

**Keywords:** Neurological practice, Neurology – pediatric

## Abstract

During the COVID-19 pandemic, the Division of Neurology at BC Children’s Hospital rapidly transitioned to almost exclusively virtual health. In April 2020, 96% of outpatient visits were done virtually (64%) or by telephone, and only 4.2% were in-person. Total clinic visit numbers were unchanged compared to previous months. Neurologists reported high satisfaction with the virtual history and overall assessment, while the physical examination was less reliable. Additional in-person visits were rarely required. Rapid, sustained adoption of virtual health is possible in a pediatric neurology setting, providing reliable care that is comparable to in-person consultations when physical distancing is necessary.

## RÉSUMÉ

**Passage rapide aux consultations virtuelles dans un service de neuropédiatrie durant la pandémie de COVID-19.**

Durant la pandémie de COVID-19, la division du service de neurologie au BC Children’s Hospital est passée rapidement aux consultations virtuelles, et ce, en quasi-totalité. En avril 2020, 96 % des consultations externes se donnaient virtuellement (64 %) ou par téléphone, et seulement 4,2 % des autres se tenaient en personne. Pourtant, le nombre total de consultations cliniques n’a pas changé par rapport à celui enregistré les mois précédents. De plus, les neurologues ont fait état d’un degré élevé de satisfaction au regard de la prise virtuelle de l’anamnèse et de l’évaluation globale, mais l’examen physique, lui, s’est révélé moins fiable. Il a rarement été nécessaire de recourir aux consultations complémentaires en personne. Ainsi, l’adoption rapide et la prestation prolongée de visites virtuelles est possible en neuropédiatrie, ce qui permet de fournir des soins de qualité, comparables à ceux donnés durant les consultations en personne lorsque des mesures de distanciation physique s’imposent.

Telehealth, the delivery of health care using electronic communication, allows patients to access health care remotely, either from a local satellite site^1^ (often referred to as telemedicine), or from home using a personal electronic device (specifically referred to as virtual health). Non-inferiority of telehealth evaluations and comparable efficacy to in-person visits has been demonstrated^2,3^ and telehealth in pediatric neurology results in high patient satisfaction, reducing time, travel, and cost.^4,5^ The importance of history and visual inspection^1,4,6^ in the clinical evaluation, especially in young children, and the concentration of pediatric neurologists in urban areas^6–8^ make telehealth well suited for pediatric neurology, though limitations in examination have restricted its use largely to follow-up visits.^9^

The COVID-19 pandemic has highlighted many advantages of virtual health, such as ability of both patient and provider to remain at home,^10^ greater convenience, and reduced cost.^11,12^ Recently, Grossman and colleagues described a successful transition to almost exclusively virtual adult neurology outpatient care during the COVID-19 pandemic.^13^ Rametta and colleagues assessed the implementation of remote telehealth services in pediatric neurology in response to the COVID-19 outbreak by comparing 2093 virtual encounters with previous in-person encounters. They found high satisfaction among providers, and a low rate of in-person encounters following the virtual visit.^14^

Many children in British Columbia live far from specialist care. In 2019, telemedicine accounted for 4.6% of total encounters within the Division of Neurology at BC Children’s Hospital (BCCH), the only large, publicly funded, academic hospital-based pediatric neurology practice in the province. Prior to the COVID crisis, 12 of the 14 pediatric neurologists in our practice had used telemedicine, while only 4 had done virtual health in a pilot study comparing virtual health to conventional telemedicine.

We assessed the impact of large-scale transition to virtual health on overall clinic visit numbers, obtained from internal booking data. We assessed physician-reported satisfaction with virtual health by means of two surveys. We hypothesized that prior experience with telehealth would facilitate a successful and widespread transition to virtual health, minimizing cancelations and maintaining clinic visit volumes.

On March 16, 2020, physical distancing restrictions were enacted at BCCH and all non-urgent outpatient in-person and telehealth visits were converted to virtual health or telephone consultation or postponed. It was agreed that all new and follow-up outpatients would be seen by virtual health, except those with a suspected acutely evolving neurological condition, medical instability, or need for in-person physical examination, who would be seen by the on-call team where possible. Virtual visits were done using Skype for Business and Zoom, which were compliant with provincial confidentiality regulations. Rapid conversion to a largely virtual health model of care required training and credentialing for the neurologists and clerical staff.

**Figure 1:**
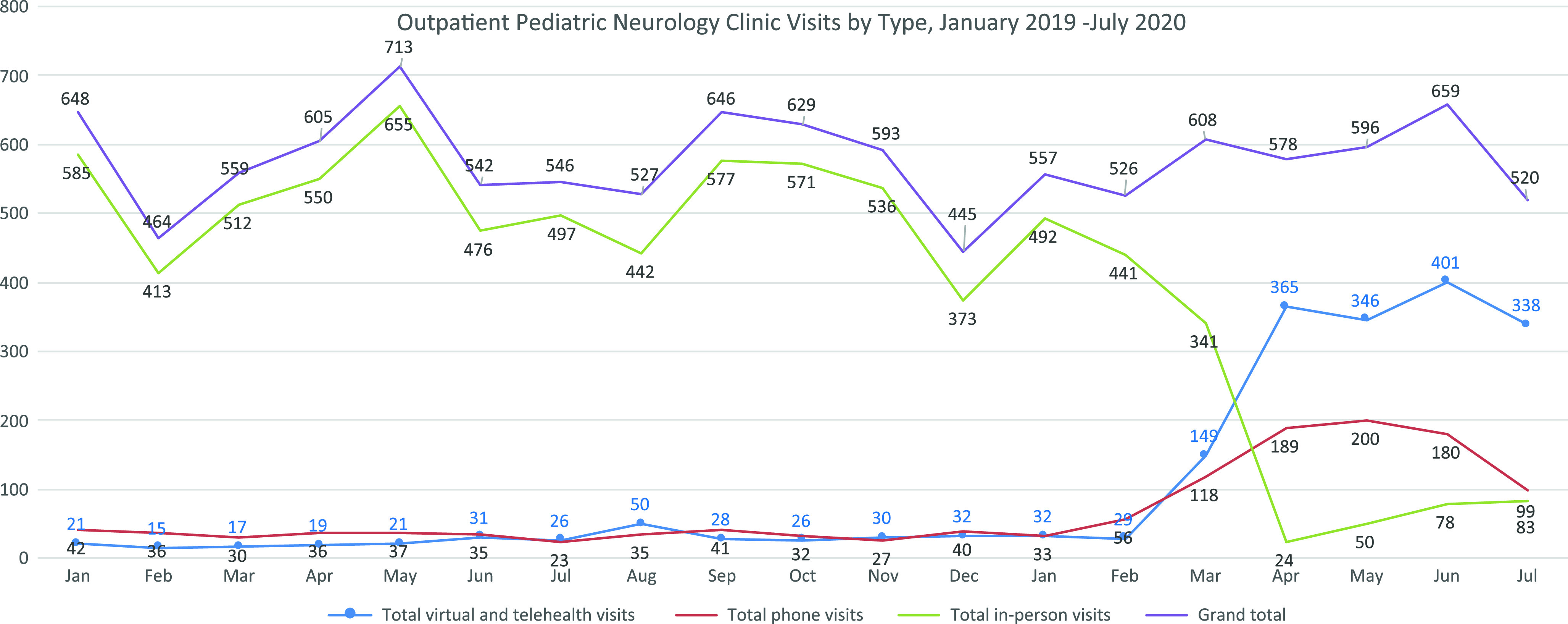
Physical distancing measures were implemented in mid-March 2020. In April 2020, virtual health visits represented 63.1% of all outpatient neurology visits. The number of virtual health visits remained consistent through July 2020, while a rebound in the number of in-person visits was largely offset by a decrease in telephone consultations.

By the end of April 2020, the first full month with physical distancing measures, the pediatric neurology clinic successfully implemented a large-scale transition to virtual health. By July, 13 (93%) pediatric neurologists had used virtual health. In April, virtual visits accounted for 63.1% (365) of visits, and in-person visits made up only 4.1% (24), with the remaining 32.8% by telephone (combined virtual and telephone consultations accounted for 95.9% of all encounters). This increase was sustained, with 61.6% of all visits between April and July done virtually (Figure 1), while increased in-person encounters in the summer months were offset by a corresponding decrease in telephone consultations.

The median number of monthly visits between March and July 2020 (596) was comparable to the median in 2019 (576). Monthly visit numbers during the pandemic were also similar to the corresponding months in 2019 (Supplementary table). New patients were seen by virtual health and overall at a rate comparable to baseline. The types of patients seen by virtual health did not differ from baseline, with no subspecialty neurology clinics reporting increased cancelations or delays due to the transition to virtual health. In some instances, such as neuromuscular clinics, challenges were noted related to the need for a complete physical examination, particularly among new patients, though visit numbers did not decrease.

Virtual health utilization was greater in neurology compared to combined medical subspecialties at BCCH, where combined virtual health and phone encounters accounted for only 64% of all visits in March and April. Medical specialities also saw a 24% decrease in overall patient visits compared to the same period in 2019.

Two surveys were distributed to the 11 neurologists who had seen patients by virtual health. These were done as part of a quality improvement initiative and ethical approval was not required. A brief post-encounter survey assessed whether the virtual encounter was an adequate substitute for an in-person visit. Sixty-nine responses were received between March 17 and April 20, each corresponding to a single virtual health visit. In 57 (83.8%) encounters, the neurologist either strongly agreed or agreed with the statement “Virtual Health was an adequate substitute for an in-person encounter” while 3 (4.4%) disagreed. Sixty-four (92.75%) patients seen would have the same follow-up plan as if they had been seen in person, while five required an additional in-person visit to obtain additional information (typically from the physical examination). The low response rate (69 of 400 visits) may make this survey prone to selection bias.

The second survey assessed patterns of use, attitudes about efficacy and safety, and satisfaction. All 11 pediatric neurologists who did virtual consultations responded to the survey. Eight (73%) neurologists did 75% or more of their total clinic encounters following the COVID-19 outbreak using virtual health, three exclusively. All respondents strongly agreed or agreed with the statement “Virtual Health allowed me to avoid postponing or cancelling clinic visits due to the COVID-19 outbreak.” Nine (82%) strongly agreed or agreed with the statement “I was satisfied with my ability to obtain an accurate history,” though only three (27%) respondents agreed “I was satisfied with my ability to obtain physical examination findings,” five (45%) disagreed, and three were neutral. Nine (82%) respondents strongly agreed or agreed that they were satisfied with their ability to form an assessment and management plan. Follow-up plans from virtual visits were similar to in-person visits; seven (64%) respondents needed to schedule an additional in-person visit following a virtual visit less than 25% of the time. All 11 respondents agreed (9 (82%) strongly agreeing) “I would recommend the use of virtual health to my colleagues.” Ten (91%) either strongly or agreed “I plan to continue using Virtual Health to assess patients even after physical distancing restrictions are relaxed.”

Perceived patient comfort (10/11 responses) and avoiding appointment cancelation (11/11 responses) were the most frequently cited advantages of virtual health. Virtual health was felt to be superior to telephone consultations for building rapport (11/11 responses), which was not surprising as most pediatric telephone consults exclude the patient. Cost- and time-savings to families were also noted as important advantages. The inability to do physical examinations in person (8/11) and technical problems (10/11) were frequently encountered limitations. The clinician survey assessed neurologists’ overall impression of virtual health at a single point following multiple encounters, which may have made this survey subject to recall bias.

Virtual health was considered an adequate substitute for in-person visits, and the good overall satisfaction with virtual health supports its suitability in pediatric neurology for new and follow-up patients. Our findings are similar to those from a recent publication by Rametta and colleagues,^14^ who compared pediatric neurology telehealth visits to in-person encounters during the COVID-19 pandemic and found high clinician satisfaction and relatively little need for repeat visits. The similar experiences between our institution, a single, urban, Canadian center serving an entire province, and a large US network with multiple satellite locations, suggest that virtual pediatric neurology care can be successfully employed across different health care systems.

Pediatric neurology-specific clinical factors likely facilitated the transition to virtual health. Many aspects of the neurological examination can be done virtually, including speech and cognition; observations of behavior; some of the cranial nerve exam; some aspects of motor examination, coordination, and gait; and key extra-neurologic features such as dysmorphology and cutaneous examination. Relatively little of outpatient pediatric neurology is procedural, and most tests or treatments are not performed in clinic. Neurophysiologic, radiologic, and genetic investigations can easily be reviewed remotely. Limitations include the inability to assess vital signs, fundoscopy, muscle weakness, tendon reflexes, tone abnormalities, sensation, and aspects of surface anatomy such as cranial sutures and fontanelles, among others. Future work should include development of guidelines for appropriate patient selection, a standardized clinical approach to the pediatric neurological virtual examination, and evaluation of patient safety. Formal assessment of patient satisfaction would be valuable.

We describe the rapid, successful, and sustained transition of a publicly funded Canadian academic pediatric neurology outpatient practice to predominantly virtual health as a result of the COVID-19 crisis, with patient volumes maintained at levels comparable to baseline despite physical distancing, and high provider satisfaction. Successful integration of virtual health to complement in-person consultation has the potential to transform the practice of pediatric neurology and improve access to care.

## Supplementary material

For supplementary material accompanying this paper visit https://doi.org/10.1017/cjn.2020.241. click here to view supplementary material
