# Supplementary material for: Rapid Implementation of Virtual Health in a Pediatric Neurology Practice During COVID-19
Source: Can J Neurol Sci. 2020 Nov 5:1–3. doi: 10.1017/cjn.2020.241 (PMC7844157; doi:10.1017/cjn.2020.241)
Supplement: Supplementary file 1 [file S0317167120002413sup001.docx]

| Supplemental Table. Pediatric Neurology clinic visits by type of visit, January 2019 - July 2020. Data for months in which physical distancing was implemented are shown in italics. There was a large increase in the number of virtual visits starting in March 2020, sustained through July 2020. A rebound in in-person visits coincided with a decrease in telephone consults. The overall percentage of new patients seen during the pandemic remained similar to baseline. The percentage of virtual visits that were new consultations was similar to or slightly higher than the overall percentage of new patients seen. | | | | | | | | | | | | |
| --- | --- | --- | --- | --- | --- | --- | --- | --- | --- | --- | --- | --- |
| Year | Month | In-person | | | Virtual and Telehealth | | | Telephone | | | Combined (all types) | |
|  |  | Visits | % New | % of total visits | Visits | % New | % of total visits | Visits | % New | % of total visits | Total | % New |
| 2019 | Jan | 585 | 20.7% | 90.3% | 21 | 23.8% | 3.2% | 42 | 0.0% | 6.5% | 648 | 21.9% |
|  | Feb | 413 | 21.3% | 89.0% | 15 | 13.3% | 3.2% | 36 | 0.0% | 7.8% | 464 | 21.8% |
|  | Mar | 512 | 20.3% | 91.6% | 17 | 23.5% | 3.0% | 30 | 0.0% | 5.4% | 559 | 23.4% |
|  | Apr | 550 | 18.2% | 90.9% | 19 | 10.5% | 3.1% | 36 | 0.0% | 6.0% | 605 | 19.8% |
|  | May | 655 | 16.6% | 91.9% | 21 | 19.0% | 2.9% | 37 | 0.0% | 5.2% | 713 | 20.6% |
|  | Jun | 476 | 17.9% | 87.8% | 31 | 6.5% | 5.7% | 35 | 0.0% | 6.5% | 542 | 20.1% |
|  | Jul | 497 | 15.5% | 91.0% | 26 | 19.2% | 4.8% | 23 | 0.0% | 4.2% | 546 | 20.7% |
|  | Aug | 442 | 20.4% | 83.9% | 50 | 14.0% | 9.5% | 35 | 0.0% | 6.6% | 527 | 23.1% |
|  | Sep | 577 | 17.9% | 89.3% | 28 | 3.6% | 4.3% | 41 | 0.0% | 6.3% | 646 | 20.7% |
|  | Oct | 571 | 19.1% | 90.8% | 26 | 3.8% | 4.1% | 32 | 0.0% | 5.1% | 629 | 22.9% |
|  | Nov | 536 | 20.5% | 90.4% | 30 | 16.7% | 5.1% | 27 | 0.0% | 4.6% | 593 | 22.9% |
|  | Dec | 373 | 19.6% | 83.8% | 32 | 9.4% | 7.2% | 40 | 0.0% | 9.0% | 445 | 18.9% |
| 2020 | Jan | 492 | 19.5% | 88.3% | 32 | 12.5% | 5.7% | 33 | 0.0% | 5.9% | 557 | 24.8% |
|  | Feb | 441 | 21.5% | 83.8% | 29 | 6.9% | 5.5% | 56 | 0.0% | 10.6% | 526 | 21.9% |
|  | *Mar* | *341* | *18.2%* | *56.1%* | *149* | *21.5%* | *24.5%* | *118* | *13.6%* | *19.4%* | *608* | *21.5%* |
|  | *Apr* | *24* | *50.0%* | *4.2%* | *365* | *24.9%* | *63.1%* | *189* | *3.2%* | *32.7%* | *578* | *18.9%* |
|  | *May* | *0* | *42.0%* | *8.4%* | *346* | *22.8%* | *58.1%* | *200* | *7.5%* | *33.6%* | *596* | *19.3%* |
|  | *Jun* | *0* | *32.1%* | *11.8%* | *401* | *22.4%* | *60.8%* | *180* | *3.9%* | *27.3%* | *659* | *18.5%* |
|  | *Jul* | *0* | *31.3%* | *16.0%* | *338* | *23.7%* | *65.0%* | *99* | *15.2%* | *19.0%* | *520* | *23.3%* |
